# Early parental vocal contact in neonatal units: rationale and clinical guidelines for implementation

**DOI:** 10.3389/fneur.2024.1441576

**Published:** 2024-10-01

**Authors:** Manuela Filippa, Pierre Kuhn

**Affiliations:** ^1^Swiss Center for Affective Sciences, Department of Psychology and Educational Sciences, University of Geneva, Geneva, Switzerland; ^2^Division of Development and Growth, Department of Pediatrics, University of Geneva, Geneva, Switzerland; ^3^Department of Neonatal Medicine, Hautepierre Hospital University Hospital, University of Strasbourg, Strasbourg, France; ^4^Centre National de la Recherche Scientifique and University of Strasbourg, Institut des Neurosciences Cellulaires et Intégratives, Strasbourg, France

**Keywords:** preterm infants, early vocal contact, maternal voice, guidelines and recommendations, early intervention

## Abstract

This paper aims to present clear and evidence-based proposals for the integration of Early Parental Vocal Contact into the clinical practices of neonatal units. In the first part, we present a comprehensive rationale exploring the ontogenesis of voice perception in both term and preterm newborns that establishes a foundational understanding. This knowledge serves as a crucial starting point for developing evidence-based auditory and multisensory interventions aimed at fostering the developmental trajectory of preterm infants. Drawing insights from neuroscience and brain development, our proposals underscore the significance of tailoring auditory environments within neonatal settings. Special attention is given to the unique needs of preterm infants, factoring in their gestational age and maturation levels. In the second part clinical guidelines for implementation are provided and healthcare professionals are supported to assist parents in modulating their vocal interactions, aligning them with the infant’s responses. Furthermore, we provide practical suggestions for engaging in discussions with parents about the content, duration, and frequency of vocal interventions. Finally, we delve into the potential roles of caregivers, parents, and health professionals within this enriched parental vocal interactional environment. Our perspective is firmly grounded in an infant and family-centered developmental care philosophy, aiming to enhance the overall well-being and the neurodevelopment of preterm infants in neonatal units.

## 1 Rationale

### 1.1 Developmental outcome and language development of preterm infants

Preterm infants, especially those born very and extremely preterm, remain at risk of neurodevelopmental impairment. They are particularly at risk for cognitive disorders (1) for delays in language development (2) and also for psychiatric disorders (3). Many medical factors account for this higher risk of altered neurodevelopmental outcome, but environmental factors during critical periods of brain development are also involved (4). During the equivalent of the third trimester of gestation, brain development is mainly sensory driven. Epigenetic factors are apparently involved in this environmental shaping of the developing brain during sensitive periods (5) as are synaptogenesis and selective elimination of synapses during early stages of brain development (6).

The postnatal environment of preterm infants in the neonatal intensive care unit (NICU) differs markedly, in different modalities (7), from the environment they should have continued to encounter *in utero*, especially in the acoustic environment (8, 9). This new hospital environment exposes preterm infants to excessive sensory stimulation (loud and unpredictable noise, high light levels and strong odors) as well as to sensory deprivation (lack of biological sensory stimuli coming from the mother, due to early separation) that can alter their well-being and may interfere with their neurodevelopment and growth (10–12). The acoustic environment in the NICU is particularly deleterious, and this loud, highly pitched, unorganized, and unpredictable noise can contribute to the neurocognitive burden of preterm birth, leading to attention deficit disorders (13), and potential alterations in early communicative skills (ref Kihn, Filippa chapitre).

Speech and language impairments are common in preterm infants. Speech is how we say sounds and words and includes sounds articulation, vocal production and fluency of speech, i.e., rhythm. Language refers to the words we use and how we use them to share ideas and get what we want. Language includes words meaning, sentence construction. Preterm infants commonly develop speech and language impairments as they mature with delays in the acquisition of expressive language, receptive language processing, and articulation, and deficits in phonological short-term memory (14). These impairments include problems with receptive language processing and expressive language production and deficits in phonological memory (2). A systematic review showed that preterm infants have lower scores on simple and complex language function tests throughout childhood (15). They exhibit decreased auditory discrimination, difficulties in maintaining active auditory attention, and also greater difficulties in vocal perception (15). Poor language outcomes of preterm infants may occur even in the absence of major disabilities, supporting a possible effect from the nosocomial (hospital) auditory environment preterm infants are exposed in the NICU. Neurosensory impairment in auditory processing is a well-known cause of speech delay in infants, highlighting the importance of auditory inputs for normal language development. However, medical factors such as intraventricular hemorrhage or periventricular leukomalacia, or other insults to the developing brain, contribute to language deficits (16).

The causes of language impairments in former preterm infants are undoubtedly multifactorial, and are associated with the extent of prematurity, neonatal illnesses, severity of illness, hearing ability, gender, language exposure in the NICU and at home, maternal educational attainment, social and environmental status of the family, and availability of early intervention services (17). Although environmental factors are not the only ones involved, the deprivation of biologically meaningful auditory stimuli in preterm infants seems especially important in leading to poor language development.

According to these observations, developmental care strategies have been designed to adapt the sensory experiences of preterm infants in the NICU to their sensory expectations.

and capabilities (18). They aimed to enhance the access of preterm infants to the voices of their own mothers. Strategies such as parent-driven language enrichment in the NICU have been shown to mitigate speech delays among preterm infants and improve their language performances (19). Recently also, it has been shown in 63 very preterm infants, with gestational age at birth below 32 weeks, that exposure to the parents’ speech was positively associated with the preference for faces over non-face patterns and with the preference for parents over unfamiliar faces at the corrected age of 7 months (20). In that bi-centric study, the median of exposure to parents during the 2 weeks recordings was 8.3 h for mothers and 1.3 h per day. This led to a calculated exposure to the parent’s speech ranging from 31,146 to 118,096 words for 2 weeks for the mothers, and from 151 to 13,440 words for the fathers. This suggest that Early Vocal Contact may support early social development in infancy.

### 1.2 Ontogenesis of voice perception

Among human voices, the maternal voice is the most prominent vocal stimulus.

accessed by full-term newborns. The specific preference of a term newborn for its own mother’s voice has been well documented (21). The existence of a prenatal imprint of the mother’s voice has been shown by other authors in full-term newborns in their first 2 h of life (22). The mother’s voice, with its rhythmic patterns, intonation, and timbre, provides a salient and familiar auditory environment for the developing fetus, which helps in forming early neural connections and auditory processing skills (23). Recognizing the significance of prenatal auditory experience is essential to better understand the profound effect that an atypical auditory environment can exert on preterm infants during their hospitalization.

Newborns exhibit a remarkable predisposition toward voices, demonstrating an innate orientation and sensitivity to auditory stimuli from the moment of birth (24). This orientation to voices is evident in their specific responsiveness to vocal sounds compared to other auditory inputs (25). From birth, infants possess the cognitive and physiological mechanisms necessary to process and interpret auditory information, including voices, within their surrounding environment (24).

Even in the early stages of development, newborns demonstrate the ability to discriminate between different voices, distinguishing familiar voices such as those of their parents or caregivers from unfamiliar voices (26).

Moreover, newborns display preferential attention toward human speech, particularly the rhythmic and melodic features inherent in language, indicating an early sensitivity to the prosodic elements of communication (27). This early orientation to voices underscores the importance of auditory stimulation and vocal interaction in promoting infants’ cognitive, linguistic, and socio-emotional development from the very beginning of life.

Preterm infants can perceive human voice, as they can exhibit positive responses to human voices (28), similar to the signs of approach described in the synactive theory of development of the NIDCAP program (29). The results of an observational study confirmed that very preterm infants differed in their physiologic responses to human speech and non-biological NICU sounds. They displayed specific autonomic responses to human voices such as significant increases in respiratory rate and decreases in heart rate without any drop in cerebral oxygenation (30). The reductions in heart rate were similar to the responses of the orienting cardiac reflex to stimuli of particular interest (31, 32) and observed in near-term fetuses exposed to vocal stimuli (33).

Preterm infants appear able to integrate fine language differences at a cortical level and have an early ability to activate brain regions involved in linguistic processing (34). A functional optical imaging study showed that preterm infants could distinguish between two syllables (“ba” and “ga”) and between two human voices (male and female), beginning at 29 weeks post menstrual age (35), indicating that their cortical circuits could process speech even at an immature stage with incomplete layers of cortical organization.

Human speech sounds are unique signals that are essential for the subsequent language development of newborns, especially those born preterm (36, 37).

Preterm infants can also specifically perceive their mothers’ voices prior to their term-corrected age. However, the degree of exposure of preterm infants to their own mothers’ voices is highly dependent on the implementation of infant and family-centered care strategies. Many studies have evaluated the responses to the mother’s voice of preterm infants and its benefit for their well-being (38). They indicate that thay can perceive their own mother’s voice, speaking the mother’s native language and using motherese infant directed speech. However, there are differences between very preterm infants and full-term newborns in recognition capabilities (39) and also in brain processing (40). Within the initial week of their life, preterm infants with a post menstrual age of 30 ± 2.5 weeks experience a decrease in heart rate when exposed to maternal sounds compared to when they are not exposed (12). This finding aligns with the observed decrease in heart rate, along with a cardiac orienting response, in foetuses with a gestational age of 32–37 weeks when they hear their mother’s voice (41). The ability to perceive his or her own mother’s voice has been also evidenced in preterm infants at around 34 weeks of corrected age, (12, 42, 43). The specific auditory activation measured with fMRI in the left superior frontal cortex of fetuses of GA 33 weeks following mother’s voice exposure (23) indicated also a possible neural basis for this response.

Preterm infants can also physiologically respond to the mother’s voice from 30 weeks PMA and repeated exposure to maternal sounds of preterm infants of mean GA 30 weeks (range, 25–32 weeks) during the first month of life may enhance the anatomical development of their primary auditory cortex (44). This suggests an adaptive and experience-dependent brain plasticity specific to maternal sounds.

### 1.3 Maternal voice, infant-directed speech and singing for infants’ development

The speech addressed to infants is vital to their development. Infant-directed speech can contribute to the early development of language by improving the recognition of native vowel sounds, enabling the identification of individual speech units, offering clues about grammar, supporting the comprehension of sentence structure, and the acquisition of new words (45, 46). More specifically, infant-directed speech, including amplified vowel sounds, enhances their clarity and facilitates infants’ perception, and it frequently includes simplified and repetitive speech patterns that assist infants in breaking down speech into meaningful segments (47). Infant-directed speech offers also linguistic clues, like as intonation and stress patterns, which aid newborns in comprehending the language’s structure (48).

Furthermore, infant-directed speech facilitates the transmission of emotional states to newborns, hence promoting emotional connection and bonding between the carer and the infant (49). Through the employment of melodic, high-pitched tones Infant-directed speech is one of the various parental vocalizations used to sustain the attachment process between carers and infants, and infants are especially responsive to it (50). Intonation is a key element in infant-directed speech, it captivates infants’ focus and it is particularly informative for preverbal infants about the speakers’ communicative intent (51). On the cognitive side, it guides infants’ attention toward pertinent stimuli in their surroundings, aiding them in concentrating on crucial information and acquiring knowledge from their environment (52). Carers can in fact efficiently direct infants’ attention to objects, events, or activities of interest by manipulating the pitch, rhythm, and intonation of their voice. In addition, infant-directed speech functions to indicate that the newborn is the intended receiver of communication, so informing the infant that they are the primary focus of the caregiver’s attention and interaction (53). This facilitates infants’ comprehension that speech is specifically aimed at them and promotes mutual involvement in social exchanges.

Infant-directed speech serves to notify infants about the adult partner’s purpose to impart new knowledge to them. This signals the caregiver’s preparedness to participate in joint attention and shared activities (54, 55). Through the utilization of infant-directed speech, carers demonstrate their readiness to offer assistance and direction to newborns as they embark on their educational path.

Overall, the parental voice has a significant and complex impact on infant development, long before the first word’s appearance. Considering the crucial significance of the parental directed voice emphasizes the need to encourage early positive parent-infant interactions and create environments abundant in maternal vocalizations to enhance optimal infant development.

### 1.4 Early vocal contact: rational and evidence

In contrast to passive stimulation techniques like recorded voice administration (44), Early Vocal Contact is a sensory and interactive intervention providing directly by parents, using infant-directed speech and songs. It facilitates a dynamic and reciprocal exchange between parents and young children (42).

**Figure 1:**
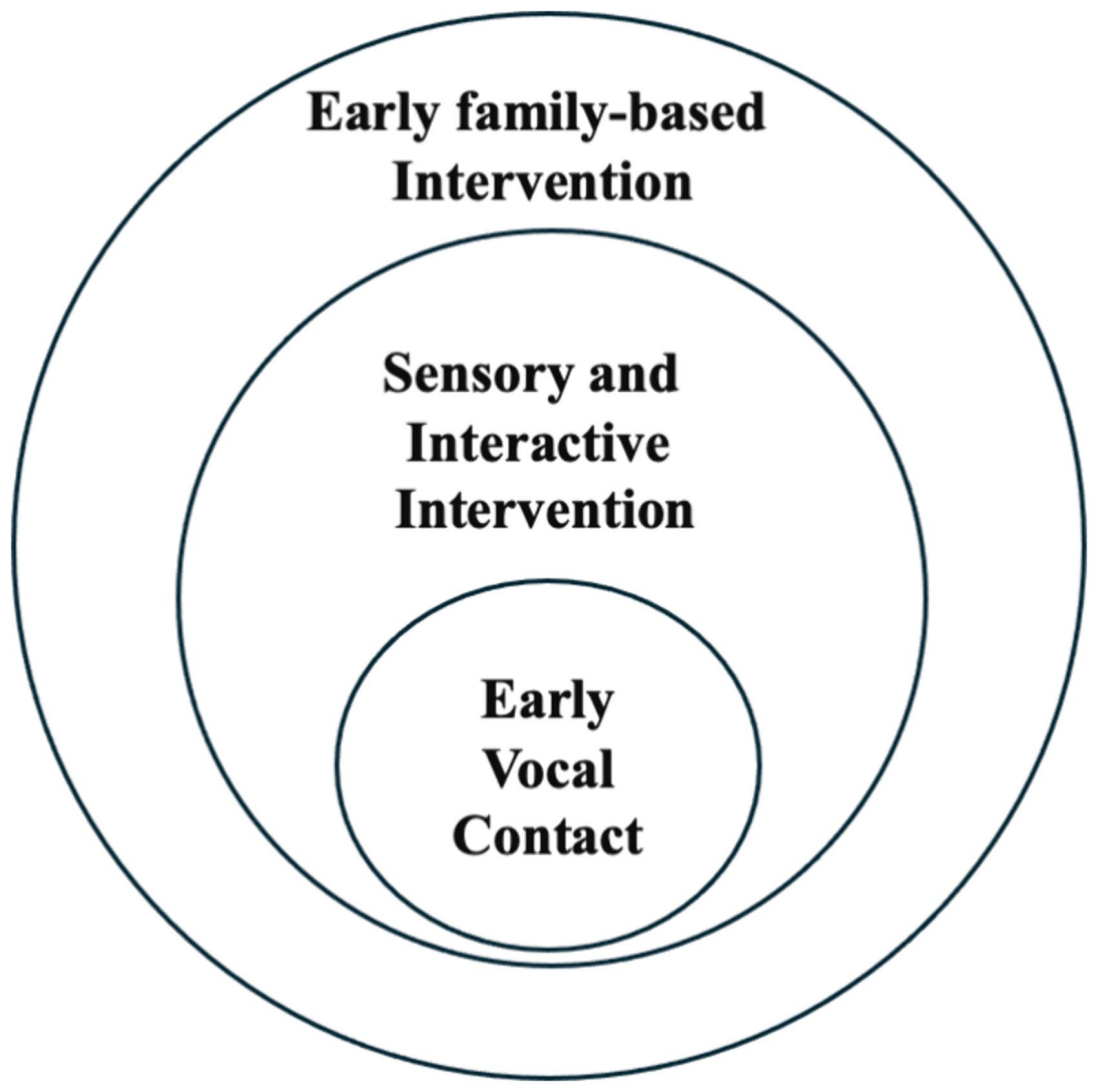
Graphical representation of the theoretical framework underlying early vocal contact.

This approach aligns with family-centered interventions in that, as depicted in Figure 1, it seeks to reactivate innate parenting abilities that might otherwise be latent in the atypical circumstance of preterm birth.

In previous research, compelling evidence of the beneficial effects of maternal singing and speech on preterm infants’ well-being was demonstrated (53).

As is the case with interventions utilizing recorded maternal voice, the evident effect of the Early Vocal Contact (EVC) is to stabilize infants’ physiological parameters, also decreasing critical cardiorespiratory events, such as apneas or bradycardias (43, 56, 57).

The maturation of the autonomous nervous system was found to be enhanced after two weeks of EVC during hospitalization, with a specific activation of the preterm infant’s parasympathetic activity (58, 59). Preterm infant-directed singing enhanced their autonomic stability (60) and decreased maternal anxiety (61). Preterm hospitalized newborns regulate their behavior in response to the acoustic properties of infant-directed speech when they hear a familiar voice addressing them (62).

During the EVC interesting infant behaviors were observed and analyzed using the System for Coding Perinatal Behavior (62). This coding system has been developed to analyze video frames by frame. The system seeks to recognize and consistently code the complex behavioral patterns in foetuses, preterm neonates, and full-term neonates from the last trimester of pregnancy to the first month following delivery. The vocalization of the mother induces premature infants to initiate eye contact and increases their self-regulatory actions, such as engaging in self-touch (62).

Finally, maternal speech and singing play a protective role against pain for preterm infants, offering a nonpharmacological intervention to alleviate pain in these vulnerable neonates. Research indicates that maternal vocalization, including both speech and singing, can effectively mitigate pain responses and enhance coping mechanisms in preterm infants during medical procedures (63).

It was recently found that maternal speech reduces pain scores and increases oxytocin levels in preterm infants undergoing painful procedures, further highlighting the positive impact of maternal vocalization in alleviating pain and promoting comfort in preterm infants (64). Overall, these findings underscore the importance of EVC in neonatal care settings, providing valuable insights into the potential mechanisms through which maternal vocalization can enhance the physiological and behavioral stability of preterm infants.

Moreover, mothers who engage in singing and talking to their premature infants during painful medical procedures experience an increase in oxytocin levels and a decrease in anxiety, indicating the potential therapeutic benefits of maternal vocalization for both mothers and infants during stressful situations (40).

At a brain level, a repeated exposure to infant-directed singing during infant’s hospitalization was positively associated with improved auditory discrimination of phonetic and emotional speech sounds in preterm infants at term age (65). Moreover, the continuous exposure to the maternal singing during skin-to-skin contact leads to larger neural responses to changes in speech sounds, interpreted as a more mature brain response to speech discrimination of preterm infants at term equivalent age (66). Interestingly, while exposed to the maternal singing preterm newborns increased their cerebral oxygenation (67). Further investigation is warranted to determine the subtle brain responses of preterm infants to maternal singing, both in terms of brain localization and brain electrical brain responses across different frequency bands.

**Figure 2:**
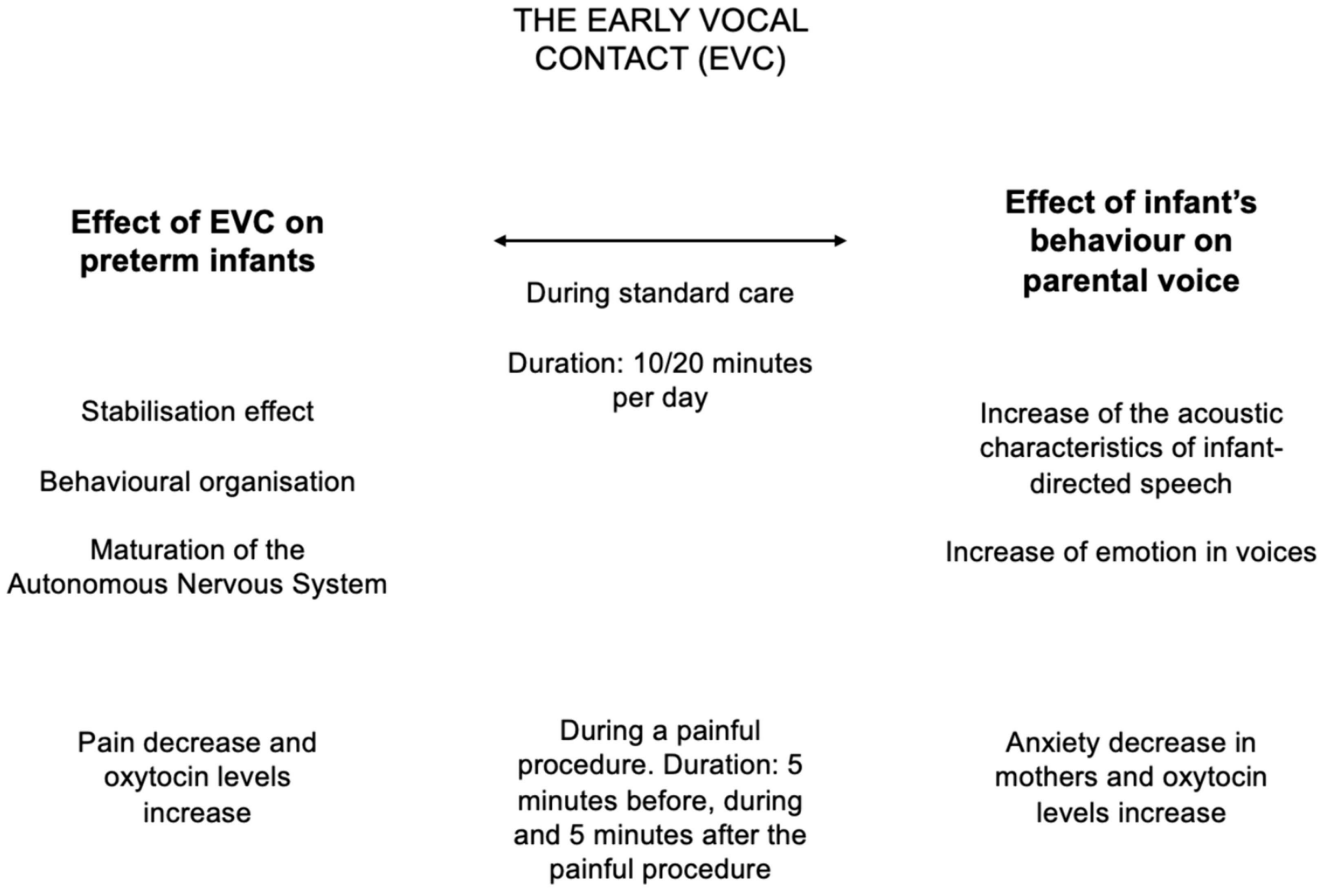
Summary of the evidence collected over the last decade on Early Vocal Contact (EVC). The effects of EVC on preterm infants at multiple levels are reported on the left column, while the effects of preterm infants behaviors on parents are detailed on the right side.

Some of the overmentioned findings are summarized in Figure 2.

In the various studies evaluating the EVC, the total duration of the mother’s voice sessions lasted from 10 to 20 min per day, three times per week, for 2 weeks or until discharge. Other research has shown a correlation between the number of words to which preterm babies are exposed and their subsequent language development, suggesting that dosage may be important and that the benefits are related to the dosage effect (Table 1).

**Table 1:** Summary of the practical recommendations for EVC.

Recommendations	For parents	For the medical team
Observe the baby’s approach and withdrawal signs	The principles of individualized care are based on observing the baby’s approach and withdrawal signs	Parents should be guided to recognize these signs and to modulate their vocal intervention accordingly (i.e., interrupt if there are withdrawal signs)
Modulate the voice	Parents’ direct voice is modulated (duration, intensity) based on the baby’s reactions	Risks of overstimulation are significantly reduced when the voice is modulated
Optimal conditions	Calm wakefulness or undifferentiated sleep state, or during transition states	- Baby in skin-to-skin contact, in a multisensory context with bone conduction of sounds - Baby in an incubator (voice 10–15 dBA above ambient background noise)
Intimacy context	Intuitive parental skills become active in an intimacy context	Ensure, as with skin-to-skin contact, an intimacy context where parents can address their baby
EVC and pain	Maternal and/or paternal voice can be useful to reduce pain	Stabilization of the baby to promote blood intake and faster return to calm in association with other non-pharmacological analgesic strategies (non-nutritive sucking, sweet solutions, skin-to-skin)
Limits	Early vocal contact with extremely or highly unstable preterm babies?	Individual responses guide the type and duration of contact (physiological responses and late or unexpected behavioral responses that are difficult to read)

Daily language exposure, beyond the EVC sessions, is likely a crucial factor in the development of preterm infants. When discussing the EVC intervention, it is important to take into account both the potential risks of language deprivation and the dangers of overstimulation. No studies have been conducted to precisely investigate the dosage effect and to evaluate whether higher exposure (for instance, 20 min every day) could provide greater benefits compared to the already evaluated dosage. These studies should be conducted to ensure that more exposure is not detrimental and could indeed be beneficial. In any case, additional exposure should be based on the evaluation of the infant’s response.

To conclude, prior research has emphasized the importance of parental vocalizations, showcasing its ability to improve the physiological stability, emotional regulation, and attachment to carers of infants. EVC offers a nurturing environment for the infant’s development, and promotes direct vocal interaction between parents and infants, which enables the exchange of emotional cues. This reciprocal intervention enhances the sensory encounters of premature infants and enables parents to engage actively in their child’s care, thereby potentially supporting long-term synchrony within the familial framework.

## 2 Proposal for good implementation in the clinical practice

The practice of EVC is based on the principles of infant and family centered developmental care (18, 68). This implies to provide a supportive sensory environment to the infant that will allow him/her to perceive infant directed speech in an intimate and secure environment supporting also parents for speech privacy (Kuhn et al., (68)). Embedded in the concept of infant centered care is also the need to individualize the care, founded above all on observation of the signs of approach and withdrawal that the baby may show in response to live vocal stimulation (69, 70). The last cornerstone of IFCDC is to have a family centered approach and to support the parents to attune the intervention to the infant’s responses and sensory expectancies.

### 2.1 Which is the ideal environment for the EVC?

First, to preserve speech privacy and audibility, the best would be to have a supportive NICU design with the infant and the parents in a private family room. It has many benefits for the infant and the parents (71) with lower background noise levels as compared to open bay NICUs or rooms with multiple bed (72, 73).

Second, an ideal environment for the infant is provided by a positive multisensory experience of the parent with intermodal redundancy (11, 74), i.e., skin to skin contact (75). Skin-to-skin contact can allow EVC to be transmitted via bone conduction, through the parent’s chest, and thus limits the loudness required for audibility. Moreover, it offers multiple benefits to the infants and the parents (75). It supports respiratory function, provides better temperature control, promotes sleep, and decreases stress in the infant, who becomes calmer and in a more stable condition to experience and benefit from EVC. Third, when the baby is in an incubator, parents should be advised to open the doors to speak to him/her. In that case, for the voice to be perceptible, it must be at a level of 10–15 dbA above the background noise (76). The background noise should be the lowest possible but noisy environment are still frequent in the NICU, especially if the infants is on nasal continuous positive airway pressure. For EVC to be natural and beneficial for both parents and baby, a context of intimacy must be provided - as with skin-to-skin contact - where adults can talk to their baby without fear of being judged or disturbing the space of other families. Thus, it is important to decrease the exposure to high sound pressure levels as much as possible whatever the nearby environment of the infant. It is therefore very important to develop and implement efficient sound abatement measures, lessening sound pressure levels and attenuating their variations near the infants. Adapting architectural design and technical equipment in the NICU to the sensory abilities of preterm infants and implementing adequate care organizations may help reduce iatrogenic noise hazards and promote access to EVC (77). The implementation of a quiet hour or the use of noise sensor light alarms has been shown efficient to sensitize medical and nursing teams and to reduce noise in the neonatal ICU (78). There has been new recommendations issued by the American Association of Pediatrics very recently that should be implemented (79) regarding the prevention of excessive noise exposure in infants.

### 2.2 Which is the infant’s optimal behavioral state for delivering the early vocal contact?

There are no studies evaluating precisely this question and data allowing to issue strong guidelines are lacking. However, the optimal conditions for delivering EVC would certainly be in a state of calm wakefulness (quiet awake) or indeterminate sleep when using the Prechtl’s classification of behavioral state (80). Preserving deep sleep and active sleep remains imperative, but when the baby’s eyes are open or when he is in a state of transition between these two states, he is more likely to pay attention to significant sensory stimuli (81). This is when parents can make their presence felt and support the transition from one state to the other. We know from previous studies that infants are more responsive to their auditory environment when they are in active sleep state (76, 82) a state in which sleep disruption may occur also after sound peaks (83). Thus, when the infant is sleeping and during skin-to-skin contact for instance, EVC could be provided but should be fully attuned to the infant’s close behavioral observation. Moreover, the results derived from a pioneering study on EVC (43), may suggest that parent talking would support quiet awake state, whereas parent singing would preserve sleep. Based on these observations, we may propose parents to provide singing at the onset of skin-to-skin contact especially during the transition from the incubator. This could help to better support the self-regulation competencies of the infant during this potentially stressful phase. Additionally in the last phase of skin-to-skin contact, parents may talk to their infants to gradually awake them, if desired to enhance social interaction and to prepare the infants for care.

### 2.3 The importance of attuning with the infant’s responses

The practice of EVC is based on the principles of individualized care and is founded above all on observation of the signs of approach and withdrawal that the baby may show in response to live vocal stimulation (69). Parents should be guided to recognize these signs and to modulate their vocal intervention accordingly. These signs may indicate destabilization or over-stimulation. Unlike the recorded voice transmitted through loudspeakers or headphones, the parents’ direct voice can be modulated, either in terms of duration or intensity, according to the baby’s behavioral and physiological reactions. The risks of over-stimulation are therefore significantly reduced compared with the administration of music or the recorded parental voice. We therefore suggest observing the baby’s behavioral and physiological reactions with the parents and individualizing the intensity and duration of vocal contact. Indeed, the gestational age of the infant also modulates its response, and the very, and even more, the extremely PI may also react more physiologically in the first weeks of life (70). These responses need to be considered and the modalities of the EVC adjusted carefully to these responses.

The effects of a EVC between parents and very premature infants with serious health complications or very immature infants are currently unknown, as most studies on the effects of direct maternal voice and, in general, on multisensory contact are limited to premature infants in stable conditions (59, 84).

The precise time of onset of EVC must be judged individually and full account must be taken of the condition of each baby and its mother or father. The very preterm newborn’s responses to intervention should guide the type and duration of EVC: late or unexpected physiological (bradycardia, apnea) and behavioral responses in preterm newborns are often difficult to read, especially by parents. Before the EVC, infant’s conditions must be stable, but they do not necessarily have to be optimal as parents and babies can both benefit from this intervention, and improve their physiological and behavioral well-being as a result of this early practice (61). According to parental reports, a gentle and soft humming sound during skin-to-skin contact was found to be the appropriate level of intensity for their extremely premature baby.

The complexity of the vocal interaction can widen with increasing gestational age. Close observation of the infant, as part of the NIDCAP program (69) may be useful to reassure the parents and the caregivers about the comfort of the infant. Future studies are warranted to address these questions and would help to define more evidenced based proposals.

### 2.4 Intuitive adaptation and synchrony: two key concepts for describing vocal communication between parents and infants

Adaptation, as it pertains to parental voices, denotes the ever-changing dynamic modulation by which parents modify key acoustical features of their vocalizations - such pitch, timbre, energy and rhythm, of their discourse in order to correspond with the developing requirements and capacities of their newborn (85). This procedure conforms to the tenets of progressive development and mirrors the cognitive and affective maturation of the infant.

Adaptation is an ongoing and gradual process that mirrors the incremental transformations that occur throughout the infant’s developmental trajectory, and age is a better predictor of changes in parental speech than socioeconomic position or other family parameters (86, 87). Carers adeptly adjust the qualities of their vocalizations in accordance with the developing cognitive and sensory capacities of the neonate as time passes, thereby customizing their communication.

The average length of adult utterances increases as the child grows older, reflecting parental adaptation to the child’s increasing language skills, and the articulation rate of the fundamental frequency and vowel duration become more similar to adult-directed speech over time, though pitch variability remains consistent (88).

The analysis of the maternal voice during EVC in the NICU, revealed an adaptation of the acoustic parameters including pitch and intensity, which undergo modulation and change in response to the baby’s eye movements or smiles during the EVC (53). Furthermore, during these newborn’s positive behavioral signals and instances of reciprocal modulation during the EVC, the maternal voice is perceived by naïve listeners as being more emotive and smiling (89). Indeed, listeners perceive the mother’s smile while speaking or singing solely through auditory cues. A reciprocal cycle is thus established between the interacting partners throughout the EVC.

In general, the observation of parental voices adapting signifies a thoughtful and accommodating method of providing care, distinguished by a profound comprehension of the infant’s physiological requirements and vulnerabilities. Carers establish a nurturing and supportive atmosphere that promotes the child’s optimal development and well-being by harmonizing with the infant’s cognitive and affective development.

### 2.5 Practical modalities: the singing repertoire, the language, singing or speaking?

In recent years of research, few elements have emerged from scientific evidence to better understand the optimal practical modalities for parents to provide Early Vocal Contact and the optimal conditions for its delivery. More specifically in the following paragraph, we will reflect on what parents could sing or say during Early Vocal Contact, on the language they should chose in early communication, on the different functions that singing and speech have during the Early Vocal Contact and on the best conditions to deliver it.

Cross-cultural regularities in infant-directed vocalizations, speech, and singing have been highlighted (90), but the consistency of the frequency, context, and functions of infant-directed singing across global field sites remains controversial. Parental singing continues to be a prevalent aspect of caregiving, which is peculiar given the substantial technological advancements that have occurred in the last 30 years (91).

However, for multiple individual and environmental reasons the hospital setting profoundly changes the intuitive and spontaneous ways parents use to interact with their newborns.

Sixty percent of mothers sang spontaneously in the NICU, according to a study examining mothers’ beliefs, thoughts, and vocal behavior, and there was no correlation found between mothers’ spontaneous vocal use and their age, level of education or parenting experience, or musical background (92).

However, little is known about their spontaneous repertoire while spontaneously singing in the NICU. Based on our prior investigation on spontaneous maternal singing in the NICU (43), it was found that popular music constituted around 40% of the repertoire, whereas traditional national and regional melodies intended for an adult constituted 32%. The proportions of lullabies and children’s music in the overall repertoire were 17 and 11%, respectively (93).

The wide variety of songs included in the selection reflects the parents’ varied musical preferences, and in contrast to initial projections, parental preference prevailed over the utilization of children-oriented repertoire, including nursery rhymes and lullabies, with the preference being for the use of familiar and pleasurable adult music.

The spontaneous contents of infant-directed speech in the NICU encompass several categories and comprehend several registers.

The use of a predetermined book can be helpful for parents gaining confidence with the art of storytelling. Initially, having a set text provides structure and eases parents into the practice. However, once parents become more comfortable talking to their babies, the predetermined text becomes unnecessary. At this stage, parents can invent new stories, responding in real-time to their babies’ cues and reactions, that they could not see while reading a book. Parents can also engage in emotional dialogs, modulating on their newborn’s state and talking about their future together at home.

Describing the future and specific aspects of daily life outside of the hospital, such as the characteristics of their room and the individuals eagerly awaiting their return home, imbues the conversation with a profound emotional content.

#### 2.5.1 Which language for the infant-directed speech?

Mothers were encouraged to sing and converse in their native tongues as part of all of our accepted protocols. This is because early vocal contact serves as a means of both affective attunement and adult-child sharing of cultural practices and meanings. Mother tongues store meaning and knowledge gathered by parents or other relatives, indicating that there is a continuous drive to transmit. Mother tongues are not just a specific language but also a communication mechanism utilized among kin (94).

Parents can serve as the link between cultural practice and meaning during hospitalization, when standards of care are crucial to the life of the infant. Following their discharge, preterm babies will be part of a larger sociocultural environment where parenting philosophies have a role in baby care. Early social connection through voice, mother tongue, and culturally based play songs and lullabies is necessary for early engagement in social and cultural activities, which might provide unique challenges for individualized care in the NICU. All the components of the adult’s cultural and linguistic legacy that influence an infant’s development are present in this early form of protoconversation.

Nonetheless, the majority of parents have already decided on the language they wish to use while speaking to their infants. Research conducted on multilingual households indicates that maintaining consistency in the first language preference enhances the overall experience with that language and the implications of that decision for language acquisition, also affecting relative input frequency (95).

Seemingly, the use of song of kin can improve comfort, safety, stability, and sleep in infants (96). The use of songs of kin, either sung by parents or by a music therapist have positive affects when compared to standard lullabies (97). The use of songs of kin resulted in a higher sucking rate, indicating a preference for well-known music and supporting the hypothesis that these songs may be performed by a parent singing or a music therapist using songs that have been passed down through the generations or that are associated with a particularly significant event. Additionally, this might encourage non-nutritive sucking as an entrainment for healthier eating habits.

#### 2.5.2 Do preterm infant-directed speech or songs in the NICU serve different purposes?

In humans, ID speech and songs are universal and fundamental communicative signals for establishing contact and attachment between parents and helpless infants needing continuous care over multiple years (98).

Acoustic similarities allow listeners belonging to different cultural contexts to correctly identify infant versus non-infant directed speech and songs (90), independently from the effects of linguistic relatedness between vocalizer and listener.

Different functions of ID speech and songs have been highlighted during infant development (99) and these differences are also evidenced by the specific context, such as soothing or play contexts (100).

Differential effects of ID speech and songs directed to preterm infants have also been highlighted, which might suggest that these two universal forms of communication aimed at the young of our species may have different functions.

One notable effect is on the stabilization of the infants; both speech and songs contribute equally to stabilization, though they modulate the infants’ states differently (43). Infant-directed speech tends to increase the awakeness in preterm infants, while during singing they tend to remain in their initial state of quiet sleep. When it comes to behavioral organization, both forms of communication support similar self-regulation processes. However, singing uniquely induces rhythmic mouth movements in the infants and speech increases non-rhythmic mouth movements (62). This is probably due to the repetitive structure of the infant-directed singing, eliciting rhythmical responses in preterm infants. Regarding emotional attribution, both speech and songs elicit more emotion during positive communicative behaviors, such as eye opening and smile, but speech is perceived to convey more emotion compared to singing (89). This difference can be explained in terms of musical structure. In songs, the melody imposes constraints on pitch variability and tempo, limiting the expressiveness that can be achieved through these elements. Consequently, speech, which is not bound by such musical constraints, allows for introducing greater variability, thereby allowing listeners to perceive more differences in emotional contents. In terms of pain management, there are differential effects on the infants’ pain and oxytocin levels, with speech having a more pronounced effect on perceived pain reduction, but this difference was not significant.

Despite these differences, both speech and singing result in similar increases in maternal oxytocin levels, with the impact of speech being more apparent.

Finally, only maternal singing modulates the heart rate variability response in preterm infants when exposed to the maternal voice, suggesting that maternal singing—but not speech—significantly sustains the parasympathetic activity of the autonomic nervous system in (40).

In conclusion, both infant-directed singing and speech should be sustained during EVC between parents and preterm infants, as they have differential effects, and they potentially serve different functions in the communication.

### 2.6 Simple recommendations on how to support mothers/fathers for the EVC

In the following lines some key recommendations to support parents in the EVC are reported:

- Creating a condition of intimacy within the hospital room.
- Sensitizing parents on the need to be attentive to the infant’s responses, as the vocal contact is a dynamic dialog and not a sensory administration of a stimulus.
- Supporting parents in reading their preterm infants’ positive and negative responses to vocal contact.
- Encouraging parents to speak during the different phases of a routine painful procedure: in the preparation phase, during the procedure, and after it.
- Sustaining the parental humming, singing, and speech in a multisensory context, such as during skin-to-skin contact.
- Helping parents pay extra attention to the most fragile preterm infants, who can have negative responses to many simultaneous sensory stimulations.

To summarize, simple and clear messages should be conveyed to parents in the NICU.

First, it should be emphasized that the primary caregiver’s ability to intuitively sing and speak addressed to their infant is fundamental because it ensures the continuity and consistency of the caregiving relationship, which is critical for the child’s long-term development and well-being. When parents take on the role of the main communicators through vocal and auditory means, they create a special bond with their child that is marked by intimacy, confidence, and emotional attachment. This connection serves as the foundation for the child’s socio-emotional growth and language development, shaping their worldview and future relationships (101, 102).

Secondly, it is important to highlight that regular vocal interactions between parents and their newborn create a richer environment, leading to long-term benefits for the child’s linguistic and socio-communicative development. This helps establish a strong foundation for future learning and social skills.

Additionally, parents should be aware that their child’s sense of security and attachment is strengthened by the emotional responsiveness in their vocalizations, acting as a protective barrier against pain, adversity, and stress.

Finally, by creating a nurturing and stimulating environment, parents can experience reduced levels of anxiety themselves. Singing and speaking to their babies is beneficial for both the babies and the parents, promoting well-being for all. It is important to note that the recommendations provided below are straightforward and practical adjustments to the care of infants that can be easily implemented by both parents and medical professionals in their daily routine.

### 2.7 Fathers, mothers and the EVC

Over the past few decades, a discernible trend has emerged wherein fathers are increasingly participating in the early care of their offspring. This shift can be attributed to evolving family dynamics and shifting societal norms. In the research literature, however, the specific effects of paternal intervention on infant development have received scant attention.

More specifically, there have been few studies on fathers’ interventions in NICUs, with the majority focusing on basic skin-to-skin contact or tactile procedures (64). These interventions have demonstrated equal overall positive impacts on both mothers and fathers in terms of infant physiological and behavioral reactions. Notably, there was also evidence of a positive impact on the fathers’ mental health, suggesting that their active involvement in caregiving can reduce stress and increase emotional well-being (64).

Including fathers as active partners in the care of their preterm newborns resulted in positive outcomes for both the infants and the fathers. The presence and involvement of fathers in the NICU have been associated with improvements in the infants’ physiological stability, such as better temperature regulation and heart rate stability, and enhanced developmental outcomes (103). Fathers who engage in skin-to-skin contact and other caregiving activities also report feeling more confident and competent in their parenting role, fostering a stronger bond with their child.

Despite these promising findings, there remains a significant gap in research exploring the full potential of paternal involvement in NICU settings. Most existing studies have been limited in scope, focusing primarily on simple, direct forms of interaction. To truly harness the benefits of paternal engagement, more research is needed to develop and evaluate innovative, multimodal, and interactive interventions. These interventions should aim to provide fathers with opportunities for positive, meaningful interaction with their preterm infants, incorporating elements such as vocal communication and multisensory stimulation. By fostering a more inclusive caregiving environment that actively involves both parents, NICUs can support the holistic development of preterm infants and strengthen family dynamics from the earliest stages of life.

To address this gap, a group of researchers conducted a study to examine the direct and contingent effects of fathers’ speech on premature infants.

The results reported by the authors indicated that paternal speech had comparable and noteworthy impacts on premature infants as maternal speech. More precisely, when the infants were exposed to both explicit and implicit paternal discourse, their levels of calm arousal increased, suggesting that their emotional and physiological states were positively impacted. This indicates that the vocal interactions of fathers contribute significantly to the well-being of their infants and the development of feelings of security and comfort.

It is noteworthy that modifications in specific acoustic attributes of the father’s voice were also detected during EVC with their PI (104). A father’s voice displayed an elevated pitch when the infant was in an agitated state of arousal; this may indicate an adaptive reaction to the infant’s emotional cues. Notwithstanding this modulation, the paternal voice tended to be more subdued in comparison to the maternal voice, underscoring possible complementary distinctions in vocal communication patterns between fathers and mothers.

In general, the results highlight the significance of paternal participation in early caregiving activities and the distinct contributions that fathers make in the context of early communication.

### 2.8 What is the role of other vocal contact providers?

Despite the growing interest from musicians, music therapists, and caregivers in providing additional sources of vocal contact, the primary aim should be to support parent-infant interaction and ensure that the infant has access to the parents’ voices. These voices are biologically significant for the infant and have been proven to support language development.

While other vocal contact providers can play a role, this paper emphasizes the preferential and primary role of parents in early vocal interactions. It is crucial for every caregiver who interacts with the baby to understand the importance of using infant-directed speech as a primary tool during routine care. This helps initiate interaction with the infant.

When parents are not in the NICU, caregivers should strive to engage in early conversational turns with infants, participating in vocal dialogs to support an earlier onset of infant’s vocalizations. This engagement fosters a more supportive and developmentally beneficial environment for the infant.

## 3 Conclusion

In this viewpoint paper, we aim to propose safe and effective ways to use EVC in everyday NICU settings. There is substantial rationale to support the dissemination of EVC practices in the broad context of infant and family-centered care. EVC serves as a crucial method to address the lack of exposure preterm infants have to adequate sensory stimuli, such as social interactions and linguistic utterances.

Implementing EVC can help normalize the number of words that preterm infants hear in the NICU, bringing it closer to the level of auditory exposure fetuses experience *in utero* (8). This increased exposure is essential, as the lack of adequate auditory stimulation is likely one of the causes of atypical language and speech perception development of preterm infants (105).

Early vocal interaction is a fundamental human practice that is particularly important to integrate in the NICU routine care, for at-risk populations, such as preterm infants.

Ensuring that caregivers and healthcare professionals understand and implement EVC strategies can lead to better outcomes for both infants and their families. Therefore, it is crucial to continue researching and advocating for the use of EVC in neonatal care units worldwide.
